# Heterogeneous material models for finite element analysis of the human mandible bone – A systematic review

**DOI:** 10.1016/j.heliyon.2024.e40668

**Published:** 2024-11-30

**Authors:** Iman Soodmand, Ann-Kristin Becker, Jan-Oliver Sass, Christopher Jabs, Maeruan Kebbach, Gesa Wanke, Michael Dau, Rainer Bader

**Affiliations:** a Research Laboratory for Biomechanics and Implant Technology, Department of Orthopaedics, Rostock University Medical Center, Rostock, Germany; b Department of Oral, Maxillofacial Plastic Surgery, Rostock University Medical Center, Rostock, Germany

**Keywords:** Personalized finite element model, Mandible, Heterogeneous mechanical properties, Hounsfield units, Density, Elastic modulus

## Abstract

Subject-specific finite element (FE) modeling of the mandible bone has recently gained attention for its higher accuracy. A critical modeling factor is including personalized material properties from medical images especially when bone quality has to be respected. However, there is no consensus on the material model for the mandible that realistically estimates the Young's modulus of the bone. Therefore, the present study aims to review FE studies employing heterogeneous material modeling of the human mandible bone, synthesizing these models, investigating their origins, and assessing their risk of bias.

A systematic review using PRISMA guidelines was conducted on publications before 1^st^ July 2024, involving PubMed, Scopus, and Web of Science. The search string considered (a) anatomical site (b) modeling strategy, and (c) metrics of interest. Two inclusion and five exclusion criteria were defined.

A review of 77 FE studies identified 12 distinct heterogeneous material models, built based on different *in vitro* or computational methodologies leading to varied performance and highly deviated range of estimated Young's modulus. They are proposed for bones from five different anatomical sites than mandible and for both trabecular and cortical bone domains. The original studies were characterized with a low to medium risk of bias.

This review assessed the current state of material modeling for subject-specific FE models in the craniomaxillofacial field. Recommendations are provided to support researchers in selecting density-modulus relationships. Future research should focus on standardizing experimental protocols, validating models through combined simulation and experimental approaches, and investigating the anisotropic behaviour of the mandible bone.

## Highlights

• Current lack of consensus on heterogeneous material models specifically for mandible.
• 12 material models for finite element analysis of human mandible are identified.
• Diversity in material models is highlighted and risk of bias for each is assessed.
• Recommendations for selecting density-modulus relationships are given.
• Future directions: standard protocols, simulation-experiment validation, anisotropic behavior.

## Graphical abstract

**Figure:**
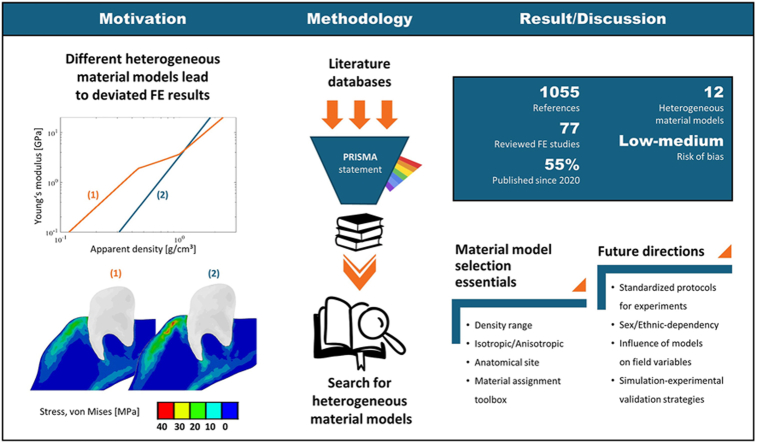

## 1 Introduction

Subject-specific finite element (FE) analysis of the mandible bone is one of the most recent advancements in biomechanics and craniomaxillofacial field and has increasingly gained attention, specifically in the field of implant design and bone-implant interaction analysis [[1], [2], [3], [4], [5]]. Alongside geometry preparation, mesh generation, and boundary conditions, the material properties are one of the main parameters influencing the accuracy of FE analysis, as they are an integral part of the stiffness matrix [2,3,6,7]. Despite the common use of homogeneous material properties for the mandible bone [7], a critical step for subject-specific FE modeling is the heterogeneous material modeling [2]. This refers to the process of assigning appropriate physical and material properties, density and Young's modulus, to different anatomical areas of the bone within a computational model. Incorporating heterogeneous material properties of bone is particularly crucial in cases where bone quality should be respected e.g., for modeling osteoporotic mandibles [8,9], age-related changes in bone properties [10], tumors-related surgery [11], bone fractures and osteosynthetic treatment [12], bone reconstruction [13,14], and temporomandibular joint replacements [15]. In this context, the primary method employed revolves around mathematical equations that correlate Hounsfield units from computed tomography (CT) [16,17] and digital imaging and communications in medicine (DICOM) databases [3,18] to both bone density and Young's modulus.

However, different material models proposing a relationship between density and Young's modulus show considerably varied material behavior. In this context, there has been no consensus on the reliability of such density-modulus relationships for the mandible bone [19], which makes comparison of different FE studies difficult [20]. Hussein et al. [1] used four different material models for mandible material properties and studied bone-implant interaction. Their comparative findings underscore the unreliability of results when employing heterogeneous bone material properties derived from different density-modulus relationships. Additionally, most of the available material models are primarily proposed for other bones e.g. femur or tibia [17,21] and may not accurately model the relationship of density and Young's modulus of the mandible bone [22].

Therefore, the present study aims to review studies employing heterogeneous material modeling of the human mandible bone, to synthesize these models, investigate their origins, and assess their quality. This may help researchers in the craniomaxillofacial field in more accurately selecting material models for constructing subject-specific FE models of the mandible bone.

## 2 Materials and methods

A systematic review was conducted on FE studies of the mandible that used heterogeneous material to model bone mechanical properties. This systematic review identified the most frequently applied material models. Furthermore, an extended search was performed to summarize and review all original studies referenced by these FE studies that proposed a density-modulus relationship. This involved examining the original studies to understand the methodologies and assumptions underlying the heterogeneous material models and assessing the risk of bias.

### 2.1 Systematic review

The systematic literature review was done according to the preferred reporting items for systematic reviews and meta-analyses (PRISMA 2020) guidelines [23].

#### 2.1.1 Literature search strategy

Literature searches were conducted on studies published prior to 11^th^ October 2023 across electronic databases, including Scopus, PubMed, and Web of Science. The search strategy was constructed by combining relevant terms and Boolean operators. These terms were derived from the titles, abstracts, and keywords indexing of 10 known and relevant studies and categorized into the following three fields of interest. This search string was examined by authors several times in a back-and-forth manner to find the optimum studies in terms of number of the studies and range of the field of studies and was used to search within article title, abstract, and keywords. The Boolean operator AND was chosen to combine the search terms and create a search string.

• Anatomical site of interest: (mandibular OR mandible OR temporomandibular OR "masticatory system")
• Modeling strategy: "finite element" AND biomech∗
• Metrics of interest: (Hounsfield OR density OR ((young∗ OR elastic) AND modu∗) OR elasticity OR heterogeneous OR ((patient OR subject) AND (specific OR personali∗)) OR ((material OR mechanical) AND propert∗))

In addition, a random and unstructured hand search was performed based on the experience of the authors. The list of references of the included articles was also explored for relevant studies. Finally, the database search was updated on 1^st^ July 2024 before the manuscript submission with the same search method.

#### 2.1.2 Inclusion and exclusion criteria

Several criteria were considered to include studies in this systematic review: (A) studies that explicitly stated their density-modulus relationship. This was done either by proposing the relationship or by clearly citing the original study describing the material model, and (B) only FE studies conducted on the mandible bone were included and considered valid for this review.

Also, five criteria were considered for excluding the studies from this review: (a) non-English studies, (b) studies with a homogenous definition for the bone, (c) studies proposing density-modulus relationship, which require extra information on bone tissue that cannot be provided by conventional CT or cone beam computed tomography scanners, (d) animal studies, because of differences in the mechanical properties of the bone between human and animal mandibles, and (e) studies with no full text available for review.

#### 2.1.3 Selection and evaluation of studies

Three independent scientists (authors A-K. B., I. S., J-O. S.) first screened the abstract and title and afterward carefully reviewed each study to ensure its comprehensiveness and eligibility and extracted the density-modulus relationships explicitly mentioned in the respective study. All relevant articles were evaluated, and those that did not fit the study's inclusion criteria were excluded. Inconsistencies over the inclusion or exclusion of a study were debated by authors until a decision was reached by consensus. In addition to the material model used, the following information was extracted from the studies: the aim of the study, material assignment toolboxes, year of publication, working group/university, and whether a full mandible bone or a block of mandible bone was used in their FE analysis.

### 2.2 Heterogeneous material models for mandible

#### 2.2.1 Data extraction

The included FE studies can be synthesized according to the employment of various density-modulus relationships. The origin of these material models was investigated, and the test conditions, determination coefficient (R^2^), and sample characteristics (bone site and domain, sizes, and geometries) were evaluated and tabulated for the original studies.

#### 2.2.2 Quality assessment

As per PRISMA guidelines, assessing the quality of the original studies, representing the study syntheses, gives insight into the study's credibility by identifying potential biases [24]. Because the original studies included two distinct types of research, separate evaluation scales, tailored to each type, were employed. The Quality Assessment Tool for *In vitro* Studies, the so-called QUIN Tool offers a standardized method for evaluating the individual risk of bias for *in vitro* studies [25]. This was carried out for all original studies that conducted *in vitro* experiments. The risk of bias for original studies based on FE simulations was assessed using the risk of bias tool for the use of finite element analysis in dentistry, the so-called ROBFEAD [26].

The aspects of both tools can be scored as either adequately/high specified = Two points, moderate specified = One point, poorly/not specified = Zero points. To provide a quantitative measure of the overall risk of bias in each study, the following equation (Equation (1)) was used for both QUIN and ROBFEAD tools [25].

(1) $$\text{Final}\;\text{score}=\frac{\text{Total}\;\text{score}\times 100}{2\times \text{number}\;\text{of}\;\text{criteria}\;\text{applicable}}$$

Two authors (A.-K. B. and I. S.) followed clear guidelines for each criteria ensuring consistency and transparency and conducted the assessments independently. Any uncertainties about missing data or disagreements were then resolved by discussion with a third author (J.-O. S.).

## 3 Results

### 3.1 Study selection

**Fig. 1:**
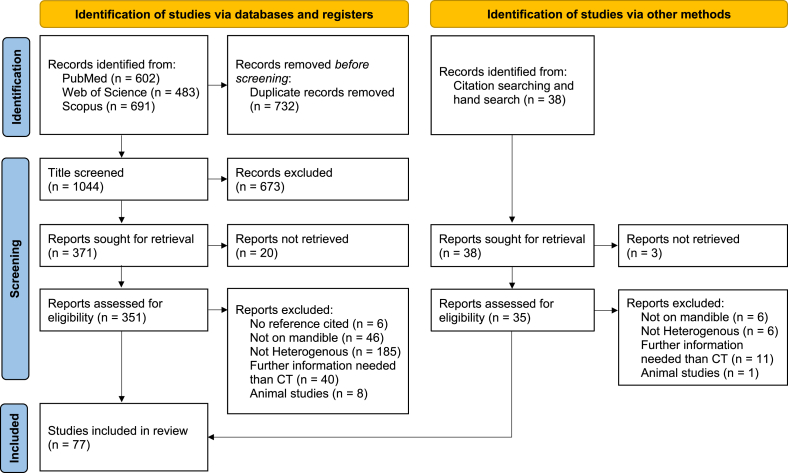
The systematic review flowchart of the study selection process (PRISMA flowchart [24]). n stands for the number of publications and CT for computed tomography.

With the described literature search strategy, 1787 studies were retrieved (Fig. 1). Following eliminating duplicates, 1055 studies underwent the abstract and title screening and 382 studies were deemed eligible for a thorough analysis. Following the full-text analysis, 77 studies were extracted that met the inclusion criteria [1,12,18,[27], [28], [29], [30], [31], [32], [33], [34], [35], [36], [37], [38], [39], [40], [41], [42], [43], [44], [45], [46], [47], [48], [49], [50], [51], [52], [53], [54], [55], [56], [57], [58], [59], [60], [61], [62], [63], [64], [65], [66], [67], [68], [69], [70], [71], [72], [73], [74], [75], [76], [77], [78], [79], [80], [81], [82], [83], [84], [85], [86], [87], [88], [89], [90], [91], [92], [93], [94], [95], [96], [97], [98], [99], [100]]. Despite our extensive efforts, 23 reports were not retrieved. While we cannot definitively assure whether these studies used homogeneous or heterogeneous material models, we note that the inability to review them does pose a risk of missing some material models relevant to our study. Unfortunately, we have no control over this matter.

Multiple studies were carried out by identical research groups, with a total of 55 authors and 31 research groups performing FE analysis using heterogeneous material models for the mandible bone. These studies were conducted from 2005 to 2024, uncovering trends in research interests over time. There are three papers published before 2010, 31 between 2010 and 2019, and 43 since 2020.

The reviewed papers cover a wide spectrum of FE analysis within the craniomaxillofacial research field. Topics include bone-implant interaction (17 studies) [1,28,33,35,36,39,46,56,59,60,71,79,80,87,88,91,92], fracture fixation (13 studies [12,29,31,48,49,[61], [62], [63],[72], [73], [74],^86^,^95^]), mandible reconstruction (10 studies [32,38,41,[51], [52], [53], [54], [55],^93^,^97^]), osteotomy techniques (eight studies [43,66,67,69,75,83,85,90]), implant-retained overdentures (six studies [34,37,47,50,81,100]) and motion/temporomandibular joint dynamics (seven studies [42,65,68,82,84,89,96]). Less frequent topics, represented by less than five publications each including dental bridges (four studies [44,45,57,58]), general investigations on healthy mandibles (three studies [18,94,98]), condylar prosthesis (three studies [27,30,77]), periodontal ligament (two studies [76,99]), influence of CT imaging on FE studies (two studies [70,78]), tumor pathology (one study [40]), and third molar tooth orientation analysis (one study [64]). Among the 77 papers, 54 studies used the full geometry of the mandible bone for FE analysis, while 23 studies employed just a block of mandible bone in the region of interest of their studies.

The process of attributing specific material properties to individual elements or nodes within a FE model based on information obtained from medical images and established material models is called “material assignment”. This procedure is referred to by various names across different studies, such as material mapping based on Hounsfield Units, and heterogeneous or inhomogeneous material modeling [101]. This can be done by various approaches [102,103] including voxel-based [104,105], element-based [106,107] or node-based methods [108,109]. There are several commercial software packages and in-house developed toolboxes available to perform the material assignment. Among the 77 reviewed studies, 20 used Mimics software package (Materialise NV, Leuven Belgium), one used mechanical finder (Research Center for Computational Mechanics, Tokyo, Japan), four used in-house developed Abaqus subroutines, and the remaining 52 studies did not clearly mention which toolboxes they used for material assignment to the mandible bone.

### 3.2 Heterogeneous material models for mandible

While reviewing the 77 FE studies and their employed density-modulus relationships, 12 different material models for the relationships between apparent (*ρ*_*app*_) or ash density (*ρ*_*ash*_) and Young's modulus (*E*) were identified and listed in Table 1 [10,15,[110], [111], [112], [113], [114], [115], [116], [117]]. The Mimics empirical default formula (first cited by Xin et al. [18]) is the most frequently cited density-modulus relationship (Equation (2)), considering solely the number of citations (18 times). However, when counting research groups using heterogenous material models, the Mimics formula is quoted only by three out of 24 research groups. Both material models proposed by Keyak et al. [113] (Equation (3)) and Rho et al. [116] (Equation (4)) emerge as the most used density-modulus relationships with each cited by six out of 31 research groups.

(2) $$E\;\left[\text{GPa}\right]=-0.3888+5.925\;\rho_{app}\;\left[g/{\text{cm}}^{3}\right]$$

$$E\;\left[\text{GPa}\right]=33.9\;\rho_{ash}^{2.2}\;\left[g/{\text{cm}}^{3}\right],\rho_{ash}\leq 0.27$$

(3) $$E\;\left[\text{GPa}\right]=5.307\;\rho_{ash}\;\left[g/{\text{cm}}^{3}\right]+0.469,0.27\;<\;\rho_{ash}\;<\;0.6$$

$$E\;\left[\text{GPa}\right]=10.2\;\rho_{ash}^{2.01}\;\left[g/{\text{cm}}^{3}\right],\rho_{ash}\geq 0.6$$

(4) $$E\;\left[\text{GPa}\right]=0.51\;\rho_{app}^{1.37}\;\left[g/{\text{cm}}^{3}\right]$$

**Table 1:**
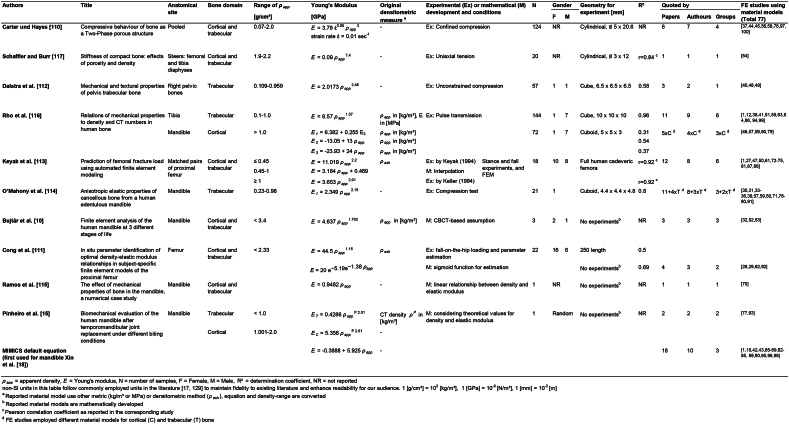
12 heterogeneous material models used in the reviewed finite element studies for the mandible.

Despite conducting an extensive literature review, the original sources for the Mimics empirical default formula could not be identified. Nevertheless, this density-modulus relationship has gained widespread recognition and become well-established since 2013 [18]. The remaining 11 material models originate from 10 studies with two employed density-modulus relationships proposed by an individual study from Rho et al. [116]. In five studies, the material model was proposed based on *in vitro* experiments, investigated with bone samples, typically through uniaxial compression or tension tests [110,112,114,116,117]. Two studies used a complete femur for their experiments and adapted previously proposed density-modulus relationships [111,113]. However, most recent studies employed a mathematical approach, where the material models were derived based on theoretical values for density and elastic modulus [15,115] or involved cone beam computed tomography-based estimations [10].

Taking into account the mathematical characteristics behind the material models, these 12 density-modulus relationships presented in Table 1 include 18 sub-functions. Among these, there are 11 power functions with exponents ranging from 1.16 to 7.4 [10,15,[110], [111], [112], [113], [114],^116^,^117^] and one sigmoid function [111]. Additionally, six linear relationships were determined [18,115,116], one of which serves as an interpolation between two power functions [113].

The origin of the density-modulus relationship varies among different bones. Two utilize data from the femur [111,113], one from the tibia [116], one from the pelvis [112], one employs a pool of various bone sites [110], and one model is derived from the femoral and tibial diaphysis of the steer [117]. Among these, five density-modulus relationships were specifically proposed based on data for the human mandible bone [10,15,[114], [115], [116]].

The original studies are conducted based on different bone domains. Among the material models, three were derived exclusively for trabecular bone [112,114,116], one for cortical bone [116], and the rest considered both bone domains, with two studies offering different sub-functions according to the density range [15,113].

Considering the constitutive modeling of the bone tissue, three material models were initially developed to accommodate the orthotropic nature of bone, addressing the directional variations by separated sub-functions [114,116]. Rho et al. [116] proposed the two density-modulus relationships as orthotropic material models for the trabecular tibia bone and cortical mandible bone. Also, O'Mahony et al. [114] proposed an orthotropic material model consisting of three directional sub-functions.

**Fig. 2:**
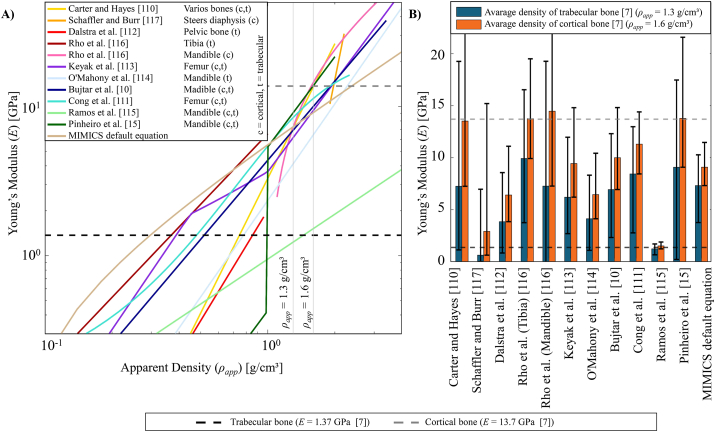
Comparison of density-modulus relationships used by finite element studies for material modelling of the human mandible bone. A: The heterogeneous material models are presented on a logarithmic scale, with the corresponding bone domain and type. Two vertical lines in light grey indicate the representative apparent density values in relation to bone domains used for the calculations in Fig. 2B B: Numerical calculation of Young's modulus for two representative apparent density values of 1.3 g/cm³ for trabecular (blue bars), and 1.6 g/cm³ for cortical (orange bars) bones [7]. In both subfigures A and B, dashed horizontal lines represent the most frequently used homogeneous Young's modulus for trabecular (1.37 GPa) and cortical (13.7 GPa) bone [7].

The differences between the material models proposed by original studies were also compared qualitatively in Fig. 2A and 2B. Prior to this comparison the material models defined in ash density (*ρ*_*ash*_) by Cong et al. [111] and Keyak et al. [113] for femur bone were converted to apparent density (*ρ*_*app*_) using the correlation *ρ*_*ash*_ = 0.6 *ρ*_*app*_ [6]. According to Schileo et al. [6], this correlation is valid for the whole density range of the femur bone. In Fig. 2A each curve presents the proposed density-modulus relationships which allows a comprehensive understanding of how different material models relate to one another. Fig. 2B shows numerical calculations of Young's modulus for two representative apparent density values.

### 3.3 Risk of bias

Table 2 summarizes the quality assessment results of the risk of bias with the QUIN and ROBFEAD tools for each original study. Studies were characterized by a low to medium risk of bias. The highest risk in the *in vitro* studies was found in Carter and Hayes [110], Dalstra et al. [112], and O'Mahony et al. [114] each with 54 % score, and the highest risk among the FE studies was found in Ramos et al. [115] with 57 % score. The density-modulus relationship known as Mimics empirical equation is characterized by an unknown/high risk of bias as the origin and methodology remained unclear.

**Table 2:**
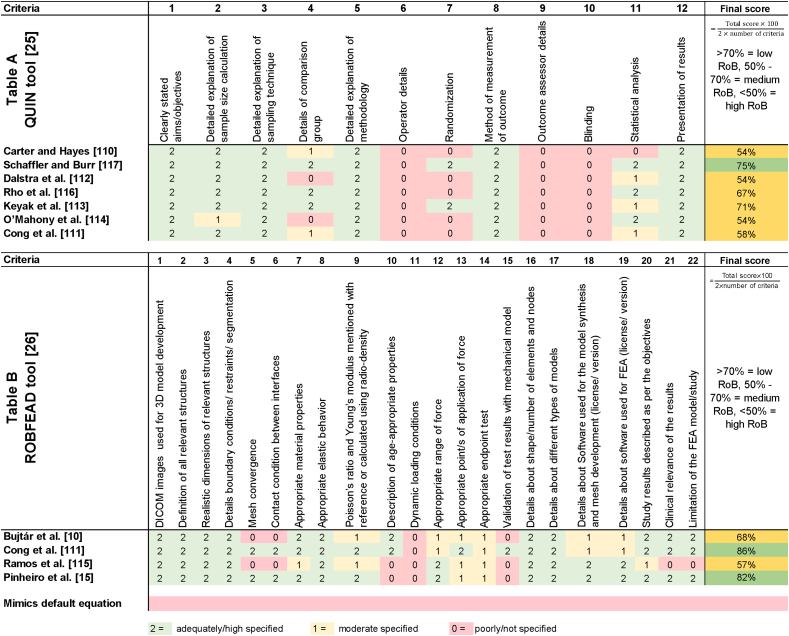
Quality assessment of original studies. **A:** Quality Assessment Tool for In vitro Studies [25], **B**: risk of bias tool for the use of finite element analysis in dentistry (ROBFEAD).[26]

## 4 Discussion

To achieve precise results in FE analysis, in addition to various other considerations, e.g. loading scenarios, boundary conditions, and the geometric details of the bone, ensuring the accurate integration of material properties stands out as a critical factor [3,5,118]. Bone is inherently a heterogeneous structure, composed of various materials arranged in complex patterns. Using heterogeneous material models in FE analysis allows for a more accurate representation of the actual mechanical behavior of bones compared to assuming homogeneity [19,21,101] and enables more realistic calculations of localized stress and strain distributions [119]. The consideration of heterogeneous material models in comparison to homogeneous models facilitates the FE models to have a greater energy dissipation [120] and a more realistic prediction of the apparent stiffness and transition between the bone domains, which avoids numerical singularities at the interface of cortical and trabecular domains [101,121]. Furthermore, integrating CT-based bone properties into FE analysis enables subject-specific modeling, allowing the comparison of individuals with different pathologies in biomechanical analysis and clinical applications.

### 4.1 Systematic review

The increasing interest in the topic of density-modulus relationships within biomedical research on the mandible is evident from the notable concentrations of publications in recent years. However, given the challenge posed by the multitude of existing material models from the literature and the increasing complexity of heterogeneous material modeling, which requires more effort, knowledge, and computational resources, yet a tendency to use homogeneous material models prevails [7]. This persists despite the multiple benefits associated with heterogeneous material modeling, as previously discussed.

The review revealed a lack of consensus among these studies regarding heterogeneous material modeling and assigning methods. Also, some studies were found which used the material model outside their intended density ranges or applied the density-modulus relationship uniformly in all directions (isotropic) despite them being primarily proposed for orthotropic materials. Such assumptions, which are common in FE analysis, may impact the accuracy of the modeling and should therefore be examined through sensitivity analysis [5]. Careful attention must be given to the correct unit conversion of the parameters of the density-modulus relationship e.g., density and elastic modulus, as this may have a significant influence on the results.

Additionally, it was observed that after selecting the appropriate heterogeneous material model, researchers used various software toolboxes for the material assignment process. Next to the toolboxes employed by the 77 reviewed studies (Mimics software package and mechanical finder) there are additional toolboxes such as Bonemat [122], Bonemapy [123], Py_bonemat_abaqus [124], Simpleware ScanIP (Synopsys Inc., United Kingdom) [125], CTPixelMapper [126], and in-house developed python plugins [127]. Based on authors experience, using a unique material model with various toolboxes, each developed using distinct assignment methods, results in considerably different Young's modulus distributions [122,128]. Notably, 52 out of the 77 studies did not specify which toolboxes or assignment strategy (node-wise or element-wise) they used, making it difficult to compare their findings.

### 4.2 Heterogeneous material models for mandible

In order to provide a better insight into the origin and reliability of density-modulus relationships and to sort the existing approaches, the second goal was to conduct an extended search identifying original studies that initially introduced heterogeneous material models. In this context, Helgason et al. [17] and Knowles et al. [129] published review studies of heterogeneous material models for human bones and discussed influences on the Young's modulus and outcomes of FE modeling [17,21]. Through the present review, 12 material models from 10 original studies were identified in total.

The absence of a consensus on the material model and the considerable large deviation among them (Fig. 2) may yield to serious implications for modeling accuracy [1]. Several factors were identified as sources of this deviation: 1) difference in study type including *in vitro* experiments, mathematical-based approach or combined simulation-experimental-based approach, 2) differences in experimental mechanical test types e.g., tension or compression test or test setups, such as constrained or uniaxial loading, and sampling rates, 3) differences in the methodology of the numerical approaches, such as different boundary condition or use case, 4) different bone samples or donors in terms of anatomical sites, geometries (cubes/cylinders or full bone), dimensions, bone domains (cortical or trabecular), the quantity of samples, sex, and even specimen-specific differences.

Irrespective of the study approaches, there is significant variation in the anatomical sites of the specimens studied in the original research. Studies by Ciarelli et al. [130] and Morgan et al. [22] revealed considerable variability in the density-modulus relationship depending on the anatomical site [22,[131], [132], [133], [134], [135]]. This becomes apparent, for instance, in samples of vertebral bones, which result in a weaker relationship between elasticity and density than the values derived from long bone samples [17].

The quantity and characteristics of bone samples differ in original studies. Despite Cong et al. [111] claiming that complete bones are more clinically relevant, most *in vitro* studies use cubic or cylindrical bone samples. Those are generally employed in a sizable cohort of samples, but it is notable that these samples often originate from a limited number of donors. For instance, Dalstra et al. [112] tested 57 samples taken from only two donor pelvic bones. This methodology restricts the ability to explore population-based differences in bone characteristics. Additionally, the potential influence of sex, age and ethnic factors on bone properties raises questions, especially when the ethnicity of the bone samples is not specified or if studies do not select a cohort of equal size in terms of their sex [135,136].

Since the trabecular and cortical bone domains cover a wide range of density, dividing the material model into sub-functions based on density ranges, known for both bone domains, is an option to better estimate the Young's modulus. Therefore, Pinheiro et al. [15] created separated sub-functions for the cortical and trabecular domains. However, this approach poses a challenge of discontinuity in material properties at the interface of bone domains, leading to complications in mesh convergence in FE analysis. An alternative strategy is provided by Keyak et al. [113], who addressed this issue by considering the transitional domain between cortical and trabecular bone within a linear sub-function, which ensures continuity at the boundaries (Fig. 2).

Additionally, several FE studies were identified that employed a density-modulus relationship proposed as orthotropic sub-functions for an isotropic behavior of the bone. For instance, all 15 FE studies that used the orthotropic material model from O'Mahony et al. [114] applied the superior-inferior sub-function as an isotropic material model for all directions. Bone anisotropy is crucial for accurately estimating the elastic modulus since the relationship between mineral content and modulus varies with the direction of loading [[137], [138], [139]].

According to the quality assessment analysis, all assessed studies have a low to moderate risk of bias (Table 2). Nevertheless, the density-modulus relationship proposed by Mimics software lacks transparency regarding its development and reliability and should therefore be used with caution.

### 4.3 Recommendations, limitations and future work

Considering the focus of this review, several recommendations can be made for using heterogeneous material models for subject-specific FE models.

– When using a material model based on *in vitro* experiments, special attention should be paid to the type of experiments and the variation in samples. In the studies in which the proposed material model is generated based on mathematical calculations, caution is required due to the potential issues with missing validation and mesh convergence, which can compromise the accuracy of the FE analysis.
– The range of applicability of the proposed density-modulus relationship in terms of bone domain and directional behavior should be respected [140] and researchers are advised to check in advance if the density-modulus relationship they intend to use is valid only for specific bone domains, density ranges or direction [3,141]. For instance, the material model from Dalstra et al. [112] is proposed only for the trabecular bone, covering the range of 0.109–0.959 g/cm^3^, and should not be used to model the bone out of this range.
– We also recommend that researchers carefully consider the material assignment method of the toolbox they employ and explicitly report it in their studies. This will enhance the reliability, comparability and reproducibility of the results for future researchers.
– Furthermore, if original studies have proposed several density-modulus relationships, for example for different anatomical sites proposed by Rho et al. [116], the specific employed material model from the original study should be cited in the scientific papers.

Although best effort was made to comprehensively gather available information, this study has some limitations. Initially designed to focus on reviewing FE analysis of the mandible using density-modulus relationships to model bone mechanical properties, it did not specifically target the material models themselves. Consequently, we may have missed some material models developed for mandible bone that have not yet been identified or cited. However, we attempted to overcome this issue through a randomized hand search. Additionally, 23 reports could not be accessed, and the inability to review them represents a potential risk of overlooking important material models that are relevant to our systematic review.

Further experiments and validations have to be conducted to: (1) provide recommendations on existing heterogeneous material models, (2) analyze the characteristics of the density-modulus relationships such as the sex-dependency, (3) investigate the influence of the employed heterogeneous material model through a comparative FE study on field variables such as stress and strain and (4) propose new models exclusively designed for both domains of the mandible bone. Prior to this, a systematic review is necessary to design a standard experimental protocol. It is also advised to use a validation approach by combining simulation and experiments such as the method presented by Cong et al. [111] to validate the material model. They estimated both power and sigmoid functions parameters, which are commonly used for bone material models, by means of optimization techniques. They iteratively updated the parameters of these functions, aiming to align the predicted femur stiffness derived from FE analysis with the experimentally determined bone stiffness acquired by them through mechanical testing [111].

Future research needs to go beyond isotropic heterogeneous material models and explore orthotropic heterogeneous material models in detail [142,143]. This approach provides a more accurate representation of the anisotropic nature of bone tissue, improving the precision of stress and strain predictions, and leading to better-informed clinical decision-making and more effective treatment planning. Furthermore, orthotropic material modeling offers the potential for even more precise subject-specific analysis by accounting for the directional dependencies of bone mechanical properties. Of the 77 reviewed scientific papers, 75 FE studies applied isotropic heterogeneous material models. While these models are more realistic than isotropic homogeneous ones, they still fail to capture the anisotropic nature of bone. Furthermore, a systematic study is required to investigate the influence of the material assignment methods and toolboxes on the results and stress distribution within the bone as inter-method differences were observed in the results produced by various material assignment toolboxes.

## 5 Conclusion

This systematic review aims to assist researchers in the craniomaxillofacial field in selecting more accurate heterogeneous material models for building subject-specific FE models of the mandible. This was achieved by conducting a systematic review on density-modulus relationships used for material modeling of the mandible bone, providing an overview of the material models that have been used and discussing the origin of the models and their quality. The lack of consensus on the optimal material model for estimating Young's modulus from mineral density for the mandible bone remains a significant challenge. The presented review highlights the variability in relationships in terms of bone domain, the methodology based on which the relationships are created, different experimental setups, isotropy or anisotropy structure, and anatomical site. This reveals the importance for researchers to carefully consider the characteristics of a relationship in accordance to the objective of their study before employing it. Future research should focus on developing new heterogeneous material models that more accurately and exclusively reflect the biomechanical properties of the mandible bone and its anisotropic structure based on a standardized experimental protocol and validating the models through methods combining simulation and experiment. Additionally, a comparative FE analysis is needed to investigate the significance of the available material models and different material assignment approaches on the resulting field variables of the bone. By addressing these challenges, researchers can improve the accuracy of subject-specific FE analysis, leading to better clinical decision-making and treatment outcomes in the craniomaxillofacial field.

## Funding

The authors are grateful to the 10.13039/501100016070ITI foundation (the International Team for Implantology) for financial support (ITI Research Grant No. 1715_2022).

## CRediT authorship contribution statement

**Iman Soodmand:** Writing – review & editing, Writing – original draft, Visualization, Validation, Resources, Project administration, Methodology, Investigation, Funding acquisition, Formal analysis, Data curation, Conceptualization. **Ann-Kristin Becker:** Writing – review & editing, Writing – original draft, Visualization, Validation, Resources, Project administration, Methodology, Investigation, Formal analysis, Data curation, Conceptualization. **Jan-Oliver Sass:** Writing – review & editing, Writing – original draft, Resources, Methodology, Investigation, Funding acquisition, Formal analysis, Conceptualization. **Christopher Jabs:** Writing – review & editing, Validation, Resources, Data curation. **Maeruan Kebbach:** Writing – review & editing, Supervision, Project administration, Funding acquisition. **Gesa Wanke:** Writing – review & editing, Resources, Methodology, Conceptualization. **Michael Dau:** Writing – review & editing, Supervision, Funding acquisition. **Rainer Bader:** Writing – review & editing, Supervision, Project administration, Funding acquisition, Conceptualization.

## Declaration of competing interest

The authors declare that they have no known competing financial interests or personal relationships that could have appeared to influence the work reported in this paper.
